# Added Value of Thin-Section Coronal DWI for Lesion Visualization in Acute Brainstem Infarction: A Retrospective Analysis

**DOI:** 10.3390/medicina62040635

**Published:** 2026-03-26

**Authors:** Alberto Negro, Mario Tortora, Ivano Palladino, Laura Gemini, Ciro Riccio, Francesco Pacchiano, Laura Lombardi, Raffaele Iaccarino, Stefano Bianco, Gianvito Pace, Simone Cepparulo, Arturo De Falco, Fabio Tortora, Giuseppe Buono, Vincenzo D’Agostino

**Affiliations:** 1 Neuroradiology Unit, Department of Neuroscience, Ospedale del Mare (ASL NA1 Centro), Via Enrico Russo, 80147 Naples, Italy; alberto.negro@hotmail.it (A.N.); laura.gemini93@gmail.com (L.G.); ciror853@gmail.com (C.R.); laura.lombardi24@gmail.com (L.L.); raffaele.iaccarino987@gmail.com (R.I.); stefano.bianco91@gmail.com (S.B.); dr.gianvito.pace@gmail.com (G.P.); dagovin79@gmail.com (V.D.); 2 Neuroradiology Unit, Department of Advanced Biomedical Sciences, University “Federico II”, Via Pansini 5, 80131 Naples, Italy; ivano.palladino@unina.it (I.P.); fpacchiano1@gmail.com (F.P.); fabio.tortota@unina.it (F.T.); 3 Neurology Unit, Department of Neuroscience, Ospedale del Mare (ASL NA1 Centro), Via Enrico Russo, 80147 Naples, Italy; cepparulo.s@gmail.com (S.C.); arturodefalco@icloud.com (A.D.F.); 4 Neuroradiology Unit, AORN “San Giuseppe Moscati”, Plesso Città Ospedaliera Contrada Amoretta, 83100 Avellino, Italy; drgbuono@gmail.com

**Keywords:** brainstem infarction, diffusion-weighted imaging, coronal DWI, lesion conspicuity, MRI, RESOLVE, medial longitudinal fasciculus, volumetric analysis

## Abstract

*Background and Objectives:* Brainstem infarctions remain challenging to identify due to their small size, complex anatomy, and known limitations of conventional axial diffusion-weighted imaging (DWI), particularly in the posterior fossa. Thin-section coronal DWI may improve lesion conspicuity by providing higher spatial resolution and an orthogonal imaging perspective. To evaluate whether 3 mm thin-section coronal DWI improves lesion visualization and delineation compared with standard 4 mm axial DWI in patients with MRI-confirmed acute brainstem infarction. *Materials and Methods:* In this retrospective single-center study, 125 consecutive patients with isolated brainstem infarction confirmed by MRI (January 2021–January 2024) were included. All patients underwent both axial and coronal DWI acquisitions. Lesions were classified by anatomical location and by the imaging plane providing better visualization (“coronal better” vs. “equal”). Lesion volumes were calculated using manual segmentation. Image interpretation was performed independently by two neuroradiologists. Interobserver agreement was assessed using Cohen’s kappa and intraclass correlation coefficient (ICC). Statistical analysis included both parametric and nonparametric tests, with confidence intervals reported. *Results:* Coronal DWI provided improved or equivalent lesion visualization in all cases. Improved visualization was most frequent in midbrain infarctions (100%) and in a subset of medullary lesions (26.7%). Lesions better visualized on coronal DWI were significantly smaller than those equally visualized (mean volume ~0.23 mL vs. ~0.55 mL, *p* < 0.0001). Twelve midbrain and eight medullary lesions were identified only on coronal DWI within the imaging protocol, all showing confirmation on ADC and/or FLAIR correlation. Interobserver agreement was substantial to excellent. *Conclusions:* Thin-section coronal DWI improves visualization and delineation of small brainstem infarctions, particularly in anatomically compact regions. These findings support its role as a complementary sequence rather than a replacement for standard axial imaging.

## 1. Introduction

Brainstem infarctions account for approximately 10% of ischemic strokes and are particularly challenging to diagnose due to their small size, anatomical complexity, and variable clinical presentation [1]. Among the brainstem structures, the pons is the most frequently affected region, followed by the medulla oblongata and the midbrain [2]. Clinical manifestations of brainstem infarcts are highly variable and may include cranial nerve palsies, sensory deficits, motor weakness or hemiparesis, vertigo, ataxia, and more specific syndromes such as medial longitudinal fasciculus (MLF) syndrome or “crossed” brainstem syndromes, reflecting the involvement of discrete nuclei or fiber tracts [1]. The subtlety and variability of these clinical signs often delay recognition, particularly when infarcts are small, emphasizing the critical need for sensitive and precise imaging techniques. Early and accurate identification is crucial, as even tiny brainstem infarcts may have important functional consequences, including impaired eye movements, dysarthria, or gait instability, which can significantly affect patient prognosis and management strategies [1,2,3,4,5].

The vascular supply of the brainstem is intricate and involves contributions from the vertebral arteries, basilar artery, anterior spinal artery, anterior inferior cerebellar arteries, posterior inferior cerebellar arteries, superior cerebellar arteries, and posterior cerebral arteries [3]. This dense and overlapping vascular network underlies both the susceptibility of the brainstem to ischemic events and the wide variability in lesion location and size. The most common etiologies of brainstem infarction include cardioembolism, small-vessel disease affecting perforating arteries, and large-vessel disease of the vertebrobasilar system [1]. Understanding the vascular anatomy and pathophysiology of these infarcts is essential not only for accurate diagnosis but also for guiding acute therapeutic decisions, such as thrombolysis or endovascular intervention [4,5].

Diffusion-weighted imaging (DWI) is the reference technique for detecting acute ischemia; however, false-negative findings are well documented in posterior circulation strokes, especially in the brainstem. These limitations are primarily related to slice thickness, susceptibility artifacts, and partial volume effects inherent to standard axial acquisitions [4,5,6,7,8,9].

Thin-section coronal DWI has been proposed as a complementary approach, offering improved spatial resolution along the craniocaudal axis and an orthogonal view of vertically oriented structures such as the corticospinal tracts, medial lemniscus, and medial longitudinal fasciculus (MLF). This may enhance lesion conspicuity, particularly for small infarcts [5,6,10,11].

## 2. Materials and Methods

### 2.1. Study Design and Population

This retrospective, single-center study included consecutive patients with MRI-confirmed isolated brainstem infarction between January 2021 and January 2024.

Due to the study design, only patients with confirmed infarction and available DWI in both planes were included. Therefore, the study does not allow estimation of diagnostic performance metrics such as sensitivity or specificity.

### 2.2. Reference Standard

The reference standard was based on a combined clinico-radiological assessment, including clinical presentation consistent with brainstem stroke; DWI hyperintensity with corresponding ADC reduction; and, FLAIR correlation when available.

### 2.3. MRI Protocol

All MRI examinations were performed on a 1.5T scanner (Amira, Siemens Healthineers, Erlangen, Germany) according to a standardized imaging protocol. Standard axial DWI was acquired with a slice thickness of 4 mm, while thin-section coronal DWI was obtained with a slice thickness of 3 mm. In addition, apparent diffusion coefficient (ADC) and fluid-attenuated inversion recovery (FLAIR) sequences were routinely acquired to provide complementary information on tissue integrity and lesion age. All diffusion-weighted sequences were performed using the RESOLVE (Readout Segmentation of Long Variable Echo-trains) technique combined with parallel imaging, which enhances spatial resolution and mitigates common limitations of echo-planar imaging in the posterior fossa, such as susceptibility artifacts, geometric distortion, and motion-related signal loss. Detailed acquisition parameters [repetition time (TR), echo time (TE), field of view, matrix size, and number of excitations, acquisition time, voxel size, slice gap, bandwidth, number of segments (EPI factor), parallel imaging factor, NEX] are provided in Table 1. This standardized approach ensured high-quality, reproducible images across all patients.

### 2.4. Image Analysis

The images were independently reviewed by two experienced neuroradiologists, each performing their assessment without consulting the other in order to ensure an unbiased evaluation. Both readers were aware of the clinical suspicion, allowing for a more focused and clinically oriented interpretation of the imaging findings.

The analysis was conducted by evaluating axial and coronal images separately, enabling a direct comparison of lesion visualization between the two planes. In particular, visualization was categorized into two main groups: “coronal better,” when coronal images provided a clearer and more defined depiction of the lesion, and “equal,” when no substantial difference in visualization quality was observed between the two planes.

Lesion boundaries were manually delineated using dedicated institutional software. This process was primarily based on the appearance of lesions on diffusion-weighted imaging (DWI), with additional support from correlation with apparent diffusion coefficient (ADC) maps and FLAIR images, in order to achieve the most accurate and consistent segmentation possible.

Finally, reproducibility was assessed. Interobserver agreement for categorical variables was evaluated using Cohen’s kappa coefficient, while volumetric agreement was quantified using the intraclass correlation coefficient (ICC), providing an objective measure of consistency between the two neuroradiologists.

### 2.5. Statistical Analysis

All statistical analyses were performed using SPSS version 31.0 (IBM Corp., Armonk, NY, USA). Lesions were classified into two groups based on their visualization on coronal versus axial DWI sequences: “coronal improved” (*n* = 23) and “equal” (*n* = 102). Lesion volumes are reported as mean ± standard deviation (SD). Normality of continuous variables was assessed using the Shapiro–Wilk test. Since lesion volumes were not normally distributed, comparisons between groups were performed using the Mann–Whitney U test, with *p*-values < 0.05 considered statistically significant.

Categorical variables, including anatomical location of lesions (midbrain, pons, medulla), were analyzed using Fisher’s exact test to examine associations between lesion classification and brainstem region. Odds ratios (ORs) with 95% confidence intervals (CIs) were calculated to quantify the relative frequency of lesion classification across regions.

Interobserver agreement was assessed by two independent neuroradiologists. Cohen’s κ was calculated for categorical evaluation (“coronal better” vs. “equal”), and the intraclass correlation coefficient (ICC) was calculated for volumetric measurements, with exact values and 95% confidence intervals reported.

Proportions of lesions confirmed by additional modalities were recorded: FLAIR changes (82.4%), ADC (100%), follow-up imaging (100%), and clinico-radiological correlation (100%). These data were used solely to support lesion identification and to ensure reproducibility in the assessment of lesion visualization.

All analyses were performed using two-sided tests. Confidence intervals for proportions were calculated using the binomial exact method, and nonparametric methods were applied where normality assumptions were not met.

## 3. Results

### 3.1. Patient Characteristics and Lesion Distribution

**Figure 1:**
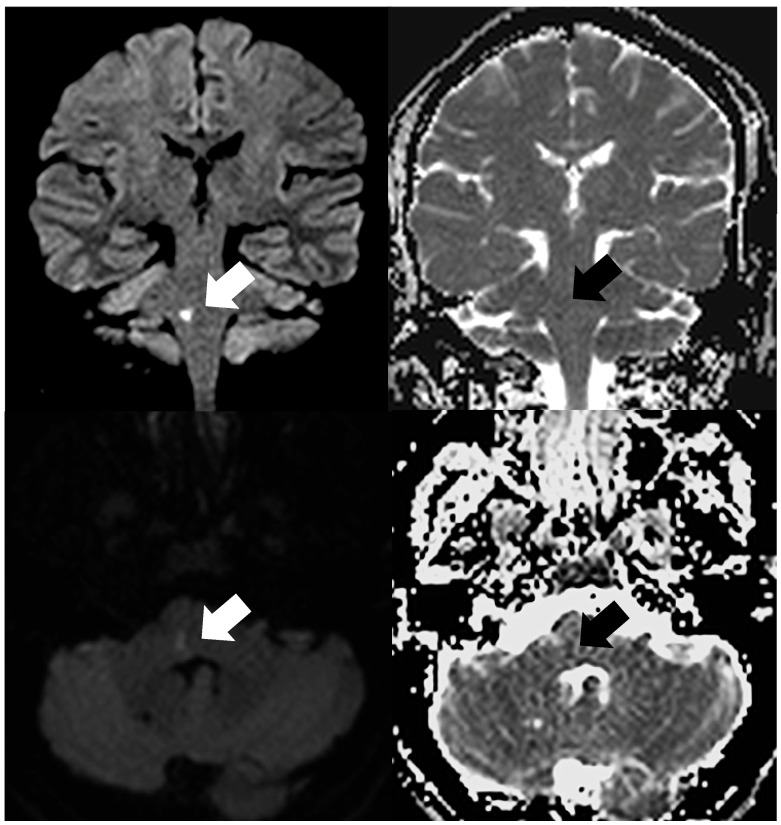
Acute ischemic lesion in the pons, specifically the right middle cerebellar peduncle. Upper row: coronal DWI (b = 1000 s/mm^2^) and corresponding ADC map; lower row: axial DWI (b = 1000 s/mm^2^) and corresponding ADC map. The lesion is indicated by a white arrow on DWI and a black arrow on ADC. Lesion conspicuity is greater on coronal images compared with axial images.

**Figure 2:**
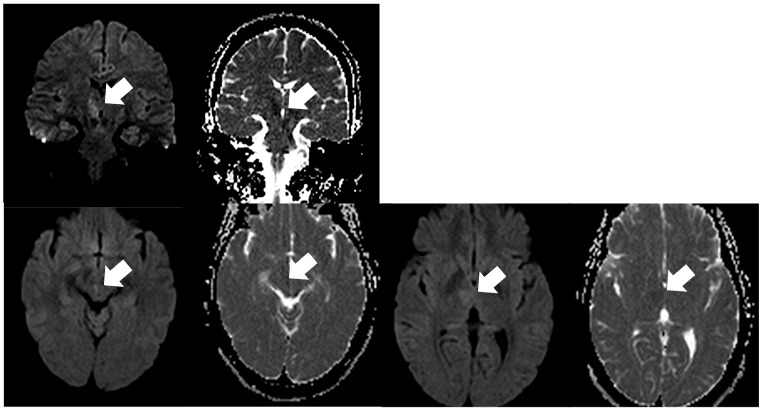
Acute ischemic lesion in the midbrain, specifically the medial longitudinal fasciculus, indicated by a white arrow. Upper row: coronal DWI (b = 1000 s/mm^2^) and corresponding ADC map; lower row: axial DWI (b = 1000 s/mm^2^) and corresponding ADC map. Lesion conspicuity is greater on coronal images compared with axial images.

A total of 125 patients were included in the study (mean age 57.1 ± 12.4 years), with a slight male predominance (56%). All lesions were confirmed using a clinico-radiological reference standard (DWI + ADC + FLAIR). FLAIR changes were observed in 103/125 (82.4%) lesions, while ADC, follow-up imaging, and clinico-radiological correlation supported 100% of lesions. Coronal-only lesions were carefully evaluated for anatomical plausibility and, when available, follow-up imaging. The anatomical distribution of brainstem infarctions was consistent with the established epidemiology of posterior circulation strokes. Most lesions were located in the pons (86/125, 68.8%), followed by the medulla oblongata (30/125, 24.0%) and the midbrain (13/125, 10.4%) (Figure 1 and Figure 2). This distribution reflects the known susceptibility of the pontine region to ischemic injury, likely related to its vascularization by small perforating branches of the basilar artery, which are particularly prone to occlusion. In contrast, infarctions involving the midbrain and medulla were less frequently observed and often presented additional diagnostic challenges due to their smaller size, more complex anatomical surroundings, and proximity to cerebrospinal fluid spaces or adjacent structures that may reduce lesion conspicuity.

### 3.2. Illustrative Cases and Anatomical Variability

Figure 1 and Figure 2 illustrate representative cases across the different brainstem regions, emphasizing not only the anatomical heterogeneity of lesion distribution but also the variability in lesion size and morphology. These examples highlight how small infarcts, particularly in more compact regions, may be less conspicuous on conventional imaging planes.

### 3.3. Anatomical Correlation and Schematic Representation

**Figure 3:**
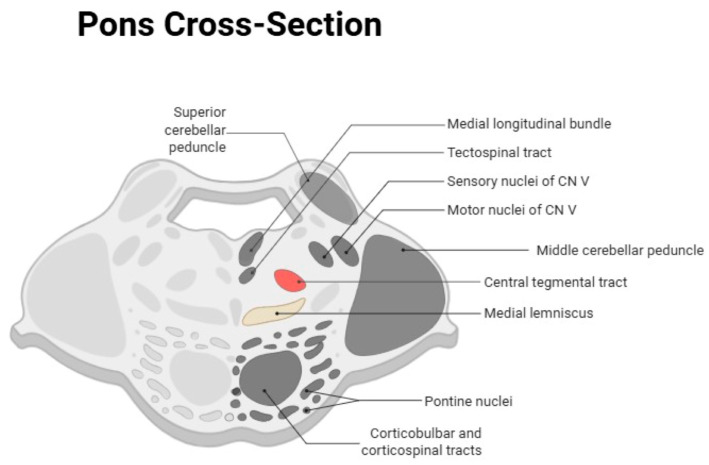
Schematic anatomical diagram of the brainstem showing key landmarks: pyramids, medial lemniscus, medial longitudinal fasciculus (MLF), and cranial nerve nuclei. Colored overlays indicate regions where coronal DWI provided the greatest lesion conspicuity.

To further support anatomical interpretation, Figure 3 provides a simplified schematic representation of the brainstem, including key structures such as the pyramids, medial lemniscus, medial longitudinal fasciculus (MLF), and cranial nerve nuclei. Superimposed color-coded regions indicate areas where coronal DWI enabled improved lesion conspicuity or visualization comparable to axial DWI. This schematic is intended to facilitate spatial understanding of lesion location, particularly for small or vertically oriented structures, and to contextualize the potential contribution of coronal acquisitions in routine imaging protocols for posterior circulation stroke.

### 3.4. Lesion Visualization According to Imaging Plane

Across the entire cohort, coronal DWI yielded lesion visualization that was either improved or comparable to that obtained with axial DWI. Notably, no lesions were identified as being more clearly depicted on axial images alone. When stratified by anatomical location, distinct patterns emerged. In the midbrain, all infarcts (13/13, 100%) demonstrated improved delineation on coronal images. This consistent finding likely reflects the compact anatomy of the midbrain and the frequent presence of small lesions aligned along the cranio-caudal axis, which may be better appreciated in the coronal plane.

In the pons, which represented the most commonly affected region, the majority of lesions (83/86, 96.5%) showed comparable visualization on both axial and coronal planes. This observation is likely attributable to the generally larger size of pontine infarcts, which facilitates their detection regardless of imaging orientation. However, a small subset of pontine lesions (3/86, 3.5%), typically smaller in size, appeared more clearly delineated on coronal images, suggesting a potential added value of this plane in selected cases.

In the medulla oblongata, a region often considered challenging due to its small size and anatomical complexity, coronal DWI provided improved visualization in 8 of 30 cases (26.7%), while the remaining lesions (22/30, 73.3%) were similarly depicted on both planes.

Fisher’s exact test confirmed a significant association between lesion location and classification (*p* < 0.0001). The odds of a lesion being in the “coronal improved” group were markedly higher in the midbrain compared with other regions (odds ratio = 125.0; 95% CI: 7.4–2100.0).

These findings indicate that coronal imaging may be particularly helpful in a subset of medullary infarctions, especially when lesions are small or located in anatomically intricate areas.

Overall, these results indicate that coronal DWI provides lesion visualization that is at least comparable to axial imaging across all brainstem regions, with a consistent pattern of improved conspicuity in smaller or anatomically compact structures. Importantly, these observations are descriptive and pertain to lesion visualization rather than diagnostic performance.

### 3.5. Lesion Volume Analysis

Quantitative analysis further supported these findings. Lesions that demonstrated improved visualization on coronal DWI were significantly smaller than those with comparable appearance on both planes. The mean lesion volume was 0.23 ± 0.12 mL in the “coronal-improved” group, compared with 0.55 ± 0.14 mL in the “comparable” group, as assessed by a Mann–Whitney U test for non-normally distributed data (*p* < 0.0001). Normality was formally tested using the Shapiro–Wilk test, confirming non-normal distribution for both groups. This suggests that the relative contribution of coronal imaging may become more relevant as lesion size decreases.

### 3.6. Lesions Identified Exclusively on Coronal DWI

A subset of infarcts was identifiable only on coronal DWI, including 12 midbrain and 8 medullary lesions. These infarctions were characterized by very small volumes (mean 0.20 ± 0.09 mL), further supporting the notion that lesion size plays a critical role in detectability across imaging planes. In four patients, coronal DWI enabled the identification of isolated infarctions involving the medial longitudinal fasciculus, a structure that is particularly small and elongated, and therefore potentially less conspicuous on axial images. These cases illustrate how anatomical orientation may influence lesion visibility.

### 3.7. Reproducibility and Interobserver Agreement

The reproducibility of imaging assessments was formally evaluated using both categorical and continuous metrics. Interobserver agreement for the qualitative evaluation of lesion visualization (“coronal better” vs. “equal”) was assessed using Cohen’s κ, which yielded a value of 0.78 (95% CI: 0.70–0.86), corresponding to a substantial level of agreement. For volumetric measurements of lesion size, the intraclass correlation coefficient (ICC) was 0.92 (95% CI: 0.88–0.95), indicating excellent agreement between the two independent neuroradiologists.

Taken together, these complementary metrics provide a robust assessment of both qualitative and quantitative reproducibility. The substantial agreement in categorical classification and the excellent consistency in volumetric measurements support the methodological soundness of the study and strengthen confidence in the reported findings.

## 4. Discussion

### 4.1. Challenges in the Detection of Brainstem Infarctions

Accurate detection of acute brainstem infarctions remains one of the most challenging tasks in clinical neuroradiology. This difficulty is largely attributable to the complex and compact anatomy of the brainstem, the small size of many ischemic lesions, and the often subtle or heterogeneous clinical presentations associated with posterior circulation strokes. In contrast to large supratentorial infarctions, brainstem lesions may involve small nuclei or fiber tracts that nonetheless produce clinically significant deficits, including cranial nerve dysfunction, ocular motility disturbances, and coordination impairment [12,13,14,15]. The clinicoradiological mismatch that may occur in these cases further underscores the importance of optimized imaging strategies.

### 4.2. Technical Considerations and Limitations of Conventional DWI

Diffusion-weighted imaging (DWI) is widely established as the cornerstone of acute stroke imaging due to its ability to detect cytotoxic edema within minutes of ischemic onset. However, conventional axial DWI sequences typically employ slice thicknesses of 4–5 mm, which may limit the visualization of very small infarcts because of partial volume effects and reduced spatial resolution, particularly in infratentorial regions [16]. These limitations are further accentuated in echo-planar imaging (EPI), where susceptibility artifacts near the skull base and cerebrospinal fluid interfaces may degrade image quality and obscure subtle lesions [17]. As a result, small infarcts in the brainstem may remain difficult to appreciate on standard axial acquisitions alone.

### 4.3. Interpretation of Study Findings

The present study investigated the contribution of thin-section (3 mm) coronal DWI in a cohort of patients with MRI-confirmed isolated brainstem infarction, focusing on lesion visualization and delineation rather than diagnostic accuracy metrics. Within this framework, coronal DWI provided lesion depiction that was either improved or comparable to axial imaging across all cases, with no instance in which axial DWI alone offered clearer visualization. This pattern was particularly evident in midbrain infarctions and in a subset of medullary lesions.

An important observation is that lesions demonstrating improved conspicuity on coronal images were significantly smaller than those with similar appearance across both planes. This finding supports the anatomical rationale that coronal orientation may mitigate partial volume effects, particularly for vertically oriented fiber tracts such as the corticospinal tracts, medial lemniscus, and medial longitudinal fasciculus (MLF). In compact anatomical regions, this may facilitate clearer depiction of lesion boundaries and spatial relationships [18]. These considerations are consistent with the notion that imaging plane orientation can influence the apparent visibility of small ischemic lesions without implying differences in diagnostic performance.

### 4.4. Comparison with Previous Literature

The potential value of multi-planar imaging in posterior circulation stroke has been increasingly explored, with several studies highlighting the complementary role of non-axial acquisitions. Felfeli et al. demonstrated that incorporating additional imaging planes can provide alternative perspectives that may facilitate the identification of infratentorial lesions, particularly in anatomically complex regions where a single-plane approach may be insufficient [17]. Their findings support the broader concept that lesion conspicuity is partly dependent on spatial orientation and that multi-planar assessment may help overcome some of the inherent limitations of axial imaging.

Similarly, prior investigations into posterior fossa stroke imaging have emphasized that orthogonal planes can improve the delineation of small or subtle infarctions, especially in cases where clinical symptoms are suggestive of brainstem involvement but axial DWI findings are inconclusive or equivocal [17]. These studies reinforce the importance of adapting imaging strategies to the anatomical and pathological characteristics of the posterior circulation.

Additional reports have explored technical refinements aimed at improving infratentorial imaging, including thinner slice acquisitions and high-resolution DWI protocols, which have been associated with better visualization of small lesions in selected contexts [18]. These approaches align conceptually with the use of thin-section coronal DWI, as both aim to reduce partial volume effects and enhance spatial resolution in regions where standard techniques may be suboptimal.

However, the literature remains heterogeneous, and not all studies have demonstrated consistent benefits from coronal imaging. Mehan et al., in a retrospective analysis, reported that the addition of coronal DWI did not significantly alter lesion detection rates and noted variability in perceived image quality across different planes [18]. Such discrepancies likely reflect differences in study design, patient populations, MRI protocols, and technical parameters, including field strength and sequence optimization.

Taken together, the available evidence suggests that coronal DWI should not be viewed as a replacement for axial imaging but rather as a complementary technique that may provide additional anatomical insight in selected scenarios. Its contribution appears to be most relevant in the evaluation of small, anatomically constrained lesions, where orientation and slice thickness play a critical role in lesion visibility.

### 4.5. Clinical Implications

From a clinical perspective, the ability to achieve clearer lesion visualization—even without direct assessment of diagnostic accuracy—may still have meaningful implications for patient management. Brainstem infarctions often present with subtle or atypical symptoms, and early imaging findings may be limited or inconclusive. In such contexts, improved lesion conspicuity can support a more confident radiological interpretation and help bridge the gap between clinical suspicion and imaging confirmation.

In particular, small infarctions involving structures such as the medial longitudinal fasciculus, paramedian pontine regions, or midbrain tegmentum are associated with well-defined clinical syndromes (e.g., internuclear ophthalmoplegia) that rely on precise anatomical localization. The improved visualization of these lesions on coronal DWI, as observed in our cohort, may therefore facilitate more accurate clinicoradiological correlation and contribute to diagnostic confidence in routine practice.

Furthermore, clearer delineation of lesion extent and location may have implications for clinical decision-making, including patient stratification, monitoring, and prognostic assessment. Although treatment decisions in acute stroke are primarily guided by clinical and temporal criteria, imaging plays a crucial role in confirming the diagnosis and excluding alternative etiologies. In this regard, the addition of a rapid coronal DWI sequence—requiring minimal extra acquisition time—may represent a practical and low-cost adjustment to existing MRI protocols.

Another relevant aspect concerns communication within the multidisciplinary team. Lesions that are more conspicuous may improve the clarity of radiological reports and facilitate discussion between neuroradiologists, neurologists, and stroke teams, particularly in complex or borderline cases. This may be especially valuable in emergency settings, where timely and accurate information is essential.

Finally, the potential benefit of coronal imaging may be greatest in cases where axial DWI findings are negative or equivocal despite persistent clinical suspicion. In such scenarios, the availability of an additional imaging plane may reduce diagnostic uncertainty and support more informed clinical decisions, even if it does not directly translate into measurable differences in diagnostic performance metrics.

Overall, while coronal DWI should not be interpreted as a standalone solution, its integration as a complementary sequence may enhance the overall robustness of MRI evaluation in suspected brainstem ischemia, particularly in selected clinical contexts.

### 4.6. Limitations

Several limitations should be acknowledged. First, the retrospective design and the inclusion of only MRI-confirmed infarctions introduce selection bias and preclude the evaluation of diagnostic performance metrics such as sensitivity, specificity, positive predictive value, and negative predictive value. Consequently, the findings are appropriately limited to descriptive assessments of lesion visualization.

Second, although image analysis was independently performed by two experienced neuroradiologists and reproducibility was formally assessed using Cohen’s kappa and the intraclass correlation coefficient (ICC), the potential influence of clinical context on image interpretation cannot be entirely excluded. Readers were aware of the clinical suspicion of brainstem infarction, which introduces the possibility of expectation bias. No specific measures to minimize systematic bias—such as randomized reading order or washout periods—were implemented. While this is acceptable given the retrospective study design, we have now explicitly emphasized this limitation to reflect its potential impact. Future studies incorporating fully blinded or randomized reading paradigms may further reduce this source of bias.

Third, the study did not include quantitative measures of image quality such as signal-to-noise ratio (SNR) or contrast-to-noise ratio (CNR). The addition of such metrics, along with advanced post-processing techniques or automated segmentation tools, could provide further insight into the technical factors influencing lesion conspicuity.

### 4.7. Future Directions

Future research may benefit from prospective study designs including both positive and negative cases, enabling a more comprehensive evaluation of the role of multi-planar DWI in clinical decision-making. The integration of advanced imaging techniques, such as high-resolution DWI sequences or reduced field-of-view acquisitions, may further improve visualization of small brainstem lesions.

In addition, emerging approaches based on artificial intelligence and machine learning offer promising opportunities. Recent studies suggest that AI-assisted analysis of multi-planar MRI datasets may enhance the identification and characterization of subtle ischemic changes, potentially supporting both automated detection and reader performance [7,19,20]. Further investigation into these technologies, particularly in the context of posterior circulation stroke, is warranted.

## 5. Conclusions

In conclusion, our retrospective analysis indicates that including a thin-section coronal DWI sequence in acute brainstem MRI protocols enhances the visualization and anatomical delineation of small ischemic lesions that may be challenging to detect on standard axial sequences alone. While this study does not address diagnostic performance in a broader clinical population, the improved conspicuity of small lesions—especially in midbrain and medullary regions—suggests that coronal DWI can provide meaningful complementary information that may support more confident neuroradiological interpretation and clinico-radiological correlation.

These findings warrant further prospective evaluation in multi-center settings that include patients with suspected posterior circulation stroke across the full spectrum of imaging outcomes. Such studies should employ robust diagnostic accuracy designs with blinded reading protocols and standardized imaging and interpretation criteria to determine whether the addition of coronal DWI ultimately improves clinical outcomes and diagnostic confidence across diverse patient populations.

## Figures and Tables

**Table 1:** The parameters of the applied DWI sequences on axial and coronal planes.

Parameter	Axial DWI	Coronal DWI
FOV (mm)	232 × 232	232 × 232
Matrix	128 × 128	128 × 128
Slice Thickness (ST, mm)	4	3
Slice Gap (mm)	0.4	0.3
Number of Slices	31	31
TR (ms)	5620	5650
TE (ms)	67	67
b-value (s/mm^2^)	0, 1000	0, 1000
Acquisition Time	2 min 12 s	2 min 20 s
Voxel Size (mm^3^)	1.8 × 1.8 × 4	1.8 × 1.8 × 3
Bandwidth (Hz/pixel)	1620	1620
Number of Segments (EPI factor)	5	5
Parallel Imaging Factor	2	2
NEX	2	2

## Data Availability

The datasets generated and/or analyzed during the current study are not publicly available due to patient confidentiality and institutional policies but are available from the corresponding author upon reasonable request.

## Abbreviations

ADC	Apparent Diffusion Coefficient
DWI	Diffusion-Weighted Imaging
FLAIR	Fluid-Attenuated Inversion Recovery
MLF	Medial Longitudinal Fasciculus
MRI	Magnetic Resonance Imaging
NEX	Number of Excitations
PACS	Picture Archiving and Communication System
RESOLVE	Readout Segmentation of Long Variable Echo-trains
TE	Echo Time
TR	Repetition Time
